# Liver metastases from colorectal cancer: regional intra-arterial treatment following failure of systemic chemotherapy

**DOI:** 10.1054/bjoc.2001.1972

**Published:** 2001-08

**Authors:** A Cyjon, M Neuman-Levin, E Rakowsky, F Greif, A Belinky, E Atar, R Hardoff, B Brenner, A Sulkes

**Affiliations:** Institute of Oncology; Departments of Diagnostic Radiology, Unit of Invasive Radiology; Surgery B; Nuclear Medicine and Sackler Faculty of Medicine, Beilinson Campus, Rabin Medical Center, Tel Aviv University, Petah Tiqva 49 100, Tel Aviv, Israel

**Keywords:** colorectal cancer, liver metastases, regional intra-arterial

## Abstract

This study was designed to determine response rate, survival and toxicity associated with combination chemotherapy delivered intra-arterially to liver in patients with hepatic metastases of colorectal origin refractory to standard systemic treatment. A total of 28 patients who failed prior systemic treatment with fluoropyrimidines received a median of 5 cycles of intra-arterial treatment consisting of 5-fluorouracil 700 mg/m^2^/d, leucovorin 120 mg/m^2^/d, and cisplatin 20 mg/m^2^/d for 5 consecutive days. Cycles were repeated at intervals of 5–6 weeks. A major response was achieved in 48% of patients: complete response in 8% and partial response in 40%. The median duration of response was 11.5 months. Median survival was 12 months at a median follow up of 12 months. On multivariate analysis, the only variables with a significant impact on survival were response to treatment and performance status. Toxicity was moderate: grades III–IV neutropenia occurred in 29% of patients. Most of the patients complained of fatigue lasting for a few days following each cycle. There were no cases of hepatobiliary toxicity. These findings indicate that regional intra-arterial treatment should be considered in selected patients with predominantly liver disease following failure of standard treatment. © 2001 Cancer Research Campaign http://www.bjcancer.com


*British Journal of Cancer* (2001) **85**(4), 504–508
© 2001 Cancer Research Campaign
doi: 10.1054/ bjoc.2001.1972, available online at http://www.idealibrary.com on IDEAL

http://www.bjcancer.com

### Liver metastases from colorectal cancer: regional intra-arterial treatment following failure of systemic chemotherapy

**A Cyjon[1], M Neuman-Levin[2], E Rakowsky[1], F Greif[3], A Belinky[2], E Atar[2], R Hardoff[4], B Brenner[1] and A Sulkes[1]**

[1]Institute of Oncology, Departments of [2]Diagnostic Radiology, Unit of Invasive Radiology, [3]Surgery B, and [4]Nuclear Medicine, Rabin Medical Center, Beilinson Campus, Petah Tiqva 49 100 and Sackler Faculty of Medicine, Tel Aviv University, Tel Aviv, Israel

**Summary** This study was designed to determine response rate, survival and toxicity associated with combination chemotherapy delivered intra-arterially to liver in patients with hepatic metastases of colorectal origin refractory to standard systemic treatment. A total of 28 patients who failed prior systemic treatment with fluoropyrimidines received a median of 5 cycles of intra-arterial treatment consisting of 5-fluorouracil 700 mg/m$^2$/d, leucovorin 120 mg/m$^2$/d, and cisplatin 20 mg/m$^2$/d for 5 consecutive days. Cycles were repeated at intervals of 5–6 weeks. A major response was achieved in 48% of patients: complete response in 8% and partial response in 40%. The median duration of response was 11.5 months. Median survival was 12 months at a median follow up of 12 months. On multivariate analysis, the only variables with a significant impact on survival were response to treatment and performance status. Toxicity was moderate: grades III–IV neutropenia occurred in 29% of patients. Most of the patients complained of fatigue lasting for a few days following each cycle. There were no cases of hepatobiliary toxicity. These findings indicate that regional intra-arterial treatment should be considered in selected patients with predominantly liver disease following failure of standard treatment. © 2001 Cancer Research Campaign http://www.bjcancer.com

**Keywords**: colorectal cancer; liver metastases; regional intra-arterial

Carcinoma of the large bowel remains the second leading cause of cancer death in the USA. Over 130 000 new cases are diagnosed each year, and over 56 000 deaths occur as a result of advanced disease (Landis et al, 1998). The liver is involved in approximately 60% of patients with advanced disease and is the sole site of initial tumour recurrence in up to 30% of patients with metastatic disease (Kemeny and Seiter, 1993).

Resection of liver metastases has become the mainstay of curative management of metastatic disease of colorectal origin (Foster and Berman, 1977), but only a minority of patients are candidates for surgery. Systemic chemotherapy with 5-fluorouracil (5-FU) modulated by leucovorin is the most frequently used palliative treatment. It is associated with a major response rate in approximately 23% of patients and a median survival of 9–12 months (Advanced Colorectal Cancer Meta-Analysis Project, 1992). Recently, a randomized trial showed that the addition of irinotecan, a camptothecin analog, to 5-FU and leucovorin significantly improves response rate and survival in patients with previously untreated metastatic disease (Douillard et al, 2000). For patients in whom progressive disease develops following initial 5-FU-based treatment, the next best available option is irinotecan, which is associated with a response rate of about 15% and moderate to severe gastrointestinal and bone marrow toxicity (Pitot et al, 1997; Rougier et al, 1997).

In view of the low response rate to 5-FU-based chemotherapy and the limited effectiveness of second-line treatment, alternatives to systemic chemotherapy have been evaluated in patients with liver metastases. The direct infusion of drugs into the hepatic arterial system takes advantage of the fact that hepatic metastases derive their blood supply fundamentally from the hepatic artery; in addition, there is evidence of nearly complete first-pass extraction of the most commonly used drugs. Thus, regional intra-arterial treatment increases the intratumoural concentration of chemotherapy while decreasing the systemic side effects. Moreover, the increase in intratumoural drug concentration achieved with this technique makes it possible to overcome the de novo and acquired cell resistance to chemotherapy (Ensminger and Gyves, 1983). Response rates ranging from 40–60% have been reported with the regional intra-arterial approach, mostly in patients without prior treatment (Kemeny et al, 1987; Chang et al, 1987; Hohn et al, 1989).

The aim of the present phase II study was to determine the survival, response rate and toxicity associated with intra-arterial chemotherapy in patients with colorectal liver metastases refractory to standard i.v. treatment with 5-FU and leucovorin. Cisplatin was added to the 5-FU and leucovorin combination on the basis of the results of in vitro and clinical studies suggesting synergism between these cytotoxic drugs, although response rate was not proven to be superior to that of 5-FU and leucovorin alone (Schabel et al, 1979; Cantrell et al, 1987; Palmeri et al, 1992; Scheithauer et al, 1991).

*Received 13 November 2000*
*Revised 27 April 2001*
*Accepted 16 May 2001*

*Correspondence to:* A Cyjon, Institute of Oncology, Assaf-Harofeh Hospital, Zrifin, Israel

### PATIENTS AND METHODS

The study population consisted of 28 patients with histologically proven adenocarcinoma of colorectal origin and evidence of non-resectable liver metastases who showed disease progression during systemic chemotherapy with 5-FU 370 mg/m$^2$/d and leucovorin 20 mg/m$^2$/d, days 1–5, every 28 d. Other inclusion criteria were:

- Bi-dimensionally measurable disease documented by abdominal computerized tomography (CT)
- Survival expectancy of more than 8 weeks
- Performance status (PS) 0–2 (ECOG scale)
- Absolute granulocyte count more than 1000/ul
- Platelet count more than 100 000/ul
- Serum bilirubin level less than 1.5 mg/dl
- Creatinine clearance over 40 ml/min.

Patients with extrahepatic metastases were entered into the study provided that the liver disease was considered to be the life-limiting factor. Two of these patients had local disease and the third patient had involvement of retroperitoneal lymph nodes. The protocol was approved by the Institutional Review Committee. All patients gave informed consent.

Baseline evaluation consisted of a complete history and physical examination as well as laboratory studies, including complete blood count and biochemical profile: liver enzymes, bilirubin, albumin, coagulation tests (prothrombin time and partial thromboplastin time), creatinine and creatinine clearance, and serum carcinoembryonic antigen (CEA) levels. All patients underwent baseline abdominal and chest CT scan, colonoscopy to rule out local recurrence, and angiography of the celiac and superior mesenteric arteries to determine hepatic arterial blood supply to the liver and patency of the portal vein. Angiography revealed a normal anatomy (type I) in 22 of the 28 patients. In 3 patients, the right hepatic artery arose from the celiac artery and the left hepatic artery from the left gastric artery (type II anatomy). In 2 patients, a replaced right hepatic artery arose from the superior mesenteric artery (type III) and in 1, the right hepatic artery arose from the superior mesenteric artery, the left hepatic artery from the left gastric artery, and the middle hepatic artery from the celiac trunk (type IV).

Initially patients received treatment via transfemoral catheterization, with placement of an intra-arterial catheter into the hepatic artery on day 1 of each cycle. Thereafter, patients who responded underwent laparotomy for placement of an internal pump (Life Port Arterial Access System, Strato Medical Corporation, Pfizer, Beverly, MA, USA) into a s.c. pocket in the upper abdominal wall. In patients with variant anatomies, treatments were administered through the different arteries. A technetium-99 macroaggregated albumin (99mTc-MAA) perfusion study was performed in all cases to confirm adequate hepatic arterial distribution of the pump effluent, to exclude extrahepatic perfusion, and to determine the pulmonary perfusion associated with intratumoural arteriovenous shunting. In 2 patients a relaparotomy was necessary to relocate a misplaced catheter.

The intra-arterial chemotherapy protocol consisted of a combination of leucovorin 120 mg/m$^2$/d over 30 min followed by 5-FU 700 mg/m$^2$/d diluted in 500 ml of normal saline with 5000 U heparin infused over 12 h, and cisplatin 20 mg/m$^2$/d diluted in 1000 cc of normal saline with 5000 U heparin infused over 12 h. This treatment was given daily for 5 consecutive days. Treatment cycles were repeated every 35–42 d. Patients were pre-medicated with ondansetron and dexamethasone to decrease emesis.

Patients were evaluated by weekly blood counts and chemistry profile. CEA levels were measured before each treatment cycle and abdominal CT scan was performed every 2 treatments to evaluate response.

Complete remission (CR) was defined as the complete disappearance of all evidence of disease on CT scan, partial remission (PR) as a more than 50% reduction in the sum of the products of the greatest perpendicular diameters of all tumour lesions on CT scan and minimal response (MR) as a 25–50% reduction in these parameters. Stabilization was defined as a reduction of less than 25% in these parameters.

Drug-related side effects were assessed at each cycle and graded according to the World Health Organization (WHO) criteria.

### Statistical methods

Survival, defined as the interval between treatment onset and death or date of last follow up, was evaluated by univariate and multivariate analysis in relation to age, gender, initial stage, PS, percentage of liver involvement as determined by CT scan, CEA level and response to treatment. The SPSS software was used to perform the Kaplan–Meier product limit method (Kaplan and Meier, 1958). To compare survival between subgroups divided according to the variables mentioned earlier and between patients with an objective response to treatment and those with stable or progressing disease, the log rank test (Peto et al, 1977) was used. Cox proportional hazards regression models (Cox, 1972) were applied for multivariate analysis. The relationship between response rate and all above-mentioned variables was evaluated with the chi-square test.

### RESULTS

The characteristics of the 28 patients in the study are shown in Table 1. The median age was 63 years (range 42–77 years). Almost half the patients were symptomatic, 9 with PS 1 and 4 with PS 2 (ECOG scale). In 43% of the patients, more than 50% of the liver was replaced by tumour. The median CEA level was 109 ng/ml (range 1.2–10 630 ng/ml). Three patients had evidence of extrahepatic disease, though the liver metastases were considered the life-limiting factor in each case. Patients received between 1 and 12 cycles of intra-arterial treatment, with a median of 5 cycles per patient. A total of 150 cycles were delivered.

Treatment was discontinued because of progression of disease

**Table 1** Baseline characteristics of study participants

| | No. patients | % |
|---|---|---|
| Gender | | |
| Male | 16 | 57 |
| Female | 12 | 43 |
| Age (years) | | |
| ≤ 65 | 16 | 57 |
| > 65 | 12 | 43 |
| Performance status | | |
| 0 | 15 | 54 |
| 1 | 9 | 32 |
| 2 | 4 | 14 |
| % Liver involvement | | |
| ≤ 50 | 16 | 57 |
| > 50 | 12 | 43 |
| CEA (ng/ml) | | |
| ≤ 100 | 14 | 50 |
| > 100 | 14 | 50 |
| Extrahepatic disease | | |
| No | 25 | 89 |
| Yes | 3 | 11 |

*© 2001 Cancer Research Campaign*

in the liver in 13 patients (46%) and extrahepatic progression in 7 patients (25%). A total of 5 patients were still receiving chemotherapy at the time of the analysis. Of the 28 patients, 3 received only one cycle of therapy and were not evaluable for response due to patient refusal to continue, poor performance status complicated by deep vein thrombosis, and bowel obstruction caused by the primary non-resected rectal tumour (1 patient each). All patients were evaluable for survival and toxicity.

### Response

Two patients (8%) achieved CR lasting 15 and 19+ months, and 10 patients (40%) achieved PR, for an overall objective response rate of 48%. None of these patients underwent any surgical procedure to resect residual tumour. Eleven patients (44%) had stable disease and in 2 patients (8%), disease progressed despite treatment. The maximal response was usually documented by the fourth cycle of treatment.

In 1 of the 3 patients with extrahepatic disease, a partial response lasting 12 months was observed. In a second patient, the disease progressed in the liver while the patient was undergoing the intra-arterial therapy and the third patient was not evaluable for response because of bowel obstruction that developed during the first cycle of intra-arterial therapy.

Median duration of response in the patients who achieved a major response was 11.5 months (range 4–19+ months). At the time of analysis at 6, 8.5, 11, 12 and 19 months, 5 patients were still in remission. The median time to progression in patients with stable disease was 6 months (range 2.5–12.5+ months).

Analysis of the relationship between response and age, gender, PS, percentage of liver involvement and CEA level showed a significant correlation only with percentage of liver involvement ($P$ = 0.029) and CEA level ($P$ = 0.027). Of patients with a tumour load of 50% or less in the liver, 67% had a major response compared to only 20% of patients with a greater tumour burden. Similarly, 69% of patients in whom baseline CEA measured 100 ng/ml or less had a major response compared to 25% of patients with higher levels.

### Survival

At a median follow up of 12 months (range 6–29 months), the median survival for all patients was 12 months (Figure 1). At the time of data analysis, 7 patients were still alive.

Univariate analysis showed that percentage of liver involvement, CEA level and response to treatment were all predictive of survival (Table 2). Patients achieving a major response and patients with stable or progressive disease had median survivals of 21 and 9 months, respectively (Figure 1). On multivariate analysis, response to treatment was the most important prognostic factor ($P$ = 0.000; risk ratio 4.2; 95% CI 1.9–9.5). PS was the only additional variable with an independent influence on survival ($P$ = 0.018, risk ratio 1.9; 95% Cl 1.1–3.4). Percentage of liver involvement and CEA level did not reach statistical significance with regard to survival (Table 3).

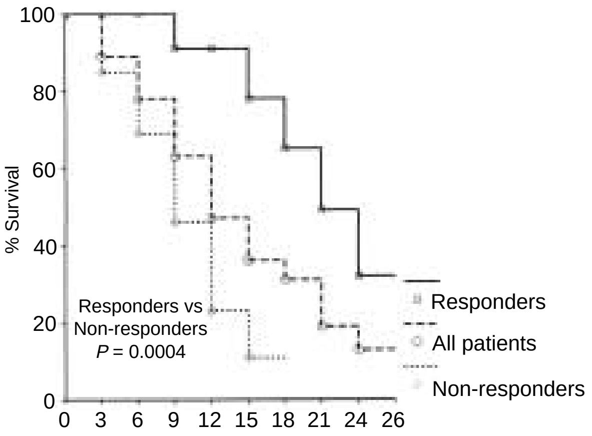

**Fig. 1** Survival.

**Table 2** Survival–univariate analysis

| Variable | No. patients | Median survival | *PP*-value |
|---|---|---|---|
| Gender | | | |
| Male | 16 | 11.0 | |
| Female | 12 | 13.0 | 0.738 |
| Age (years) | | | |
| ≤ 65 | 16 | 9.5 | |
| > 65 | 12 | 13.0 | 0.809 |
| Performance status | | | |
| 0 | 15 | 13.0 | |
| 1–2 | 13 | 9.0 | 0.159 |
| % Liver involvement | | | |
| ≤ 50 | 16 | 18.0 | |
| > 50 | 12 | 8.0 | 0.003 |
| CEA (ng/ml) | | | |
| ≤ 100 | 14 | 18.0 | |
| > 100 | 14 | 9.0 | 0.028 |
| Response* | | | |
| CR/PR | 12 | 21.0 | |
| Stable | 13 | 9.0 | 0.0004 |

*Evaluable for response: 25/28 patients.

**Table 3** Survival–multivariate analysis

| Variable | *P*-value | Risk ratio (95% CI) |
|---|---|---|
| Gender | 0.710 | NS |
| Age | 0.093 | NS |
| Performance status | 0.017 | 1.9 (1.1–3.4) |
| % Liver involvement | 0.730 | NS |
| CEA | 0.576 | NS |
| Response | 0.000 | 4.2 (1.9–9.5) |

NS = not significant.

### Toxicity

Fatigue lasting for several days was an almost universal complaint associated with treatment. Generally, patients who were fully active before treatment were able to resume their activities within a few days after each cycle. In most patients, lactic dehydrogenase, alkaline phosphatase and alanine aminotransferase levels showed a marked elevation, up to 3 times the baseline level, during treatment

© 2001 Cancer Research Campaign and for 1–2 weeks thereafter, and then reverted to baseline.

The only significant toxicity, as summarized in Table 4, was neutropenia grades III and IV, observed in 8 patients (29%). A total of 5 of these patients, (18%) were hospitalized for 6 episodes of neutropenic fever, and all recovered with parenteral antibiotics. Thrombocytopenia grades III and IV occurred in 5 patients (18%), but was not associated with bleeding episodes. Grades III and IV gastrointestinal side effects, such as diarrhoea and stomatitis, were noted in only 3 patients (11%).

Grade II neurotoxicity requiring discontinuation of cisplatin was observed in 5 patients (18%); all continued intra-arterial treatment with 5-FU and leucovorin. In 1 patient, treatment was stopped because of hepatic artery thrombosis. No clinically significant impairment in renal function was noted. There was no evident relation between severe side effects and either performance status or percentage of liver involvement. However, there were only 4 patients with PS 2 in our series. There were no treatment-related deaths in the study group.

**Table 4** Toxicity

| | No. patients ($n$ = 28) | No. cycles ($n$ = 150) |
|---|---|---|
| Neutropenia (grade III–IV) | 8 (29%) | 12 (8%) |
| Neutropenic fever | 5 (18%) | 6 (4%) |
| Thrombocytopenia (grade III–IV) | 5 (18%) | 5 (3%) |
| Diarrhoea (grade III–IV) | 2 (7%) | 3 (2%) |
| Stomatitis (grade III–IV) | 1 (4%) | 1 (1%) |

### DISCUSSION

Phase III studies of patients with metastatic liver disease of colorectal origin have consistently yielded higher response rates for first-line therapy with the direct hepatic approach than with systemic therapy. In addition, two meta-analyses have revealed a modest but statistically significant advantage in survival for patients receiving intra-arterial therapy (Harmantas et al, 1996; Meta-Analysis Group in Cancer, 1996). Nevertheless, since intra-arterial chemotherapy requires unique skills and resources and may be accompanied by specific toxicities, it has not been widely accepted either as a first-line or second-line treatment after failure of systemic fluoropyrimidines. Most patients with refractory disease are treated today with systemic irinotecan (CPT-11).

In the present study, patients with disease refractory to i.v. 5-FU and leucovorin were given intra-arterial therapy. A response rate of 48% and a median survival of 12 months were achieved. These results compare favourably with those of other investigators treating similar patients with different intra-arterial combinations. In a subgroup of 29 previously treated patients reported by Kemeny et al (1994), a response rate of 52% and a median survival of 13.5 months were attained with intra-arterial fluorodeoxyuridine (FUDR) and leucovorin. Patt et al (1997) reported a response rate of 33% and a median survival of 15 months in 48 previously treated patients receiving intra-arterial 5-FU, leucovorin and interferon. The small difference in results between these studies and ours is most probably attributable to the small number of patients in each study and the different distribution of known predictive factors, such as the extent of liver involvement. In our patients, the median liver involvement was 50%, whereas in the series of Kemeny et al (1994) and Patt et al (1997), it was 40% and less than 25%, respectively. The liver was the first site of failure in 46% of our patients, followed by extrahepatic failure in 25%. Similar rates were reported by the other authors: 64% and 29% (Kemeny et al, 1994) and 48% and 33% (Patt et al, 1997), respectively. The response rates of 33–52% and median survival of 12–15 months achieved with intra-arterial chemotherapy in patients with disease refractory to systemic fluoropyrimidines also compare favourably with the reported response rates of 13% and 18% and median survival of 8–11 months for systemic second-line irinotecan (Pitot et al, 1997; Rougier et al, 1997). Since our study was not a comparative one, we cannot exclude the possibility that similar results could be achieved with 5-FU and leucovorin alone.

In our series, treatment was relatively well tolerated. The most common complaint was fatigue, which resolved within a few days. Biliary toxicity, known to be the limiting factor associated with intra-arterial fluorodeoxyuridine administration (Kemeny and Ron, 1999), was not observed in any of our patients.

Despite the lack of randomized studies comparing intra-arterial with systemic chemotherapy for patients with fluoropyrimidine-refractory disease, the consistently higher response rates associated with the regional approach suggest that this modality should be considered in selected patients with predominant liver disease. Considering the failure rates of 25–33% in extrahepatic sites in patients receiving intra-arterial treatment, combining regional therapy with systemic therapy seems to be a rational approach. However, this strategy cannot be expected to play a significant role in improving results due to the low response rates associated with systemic administration of the currently available drugs. At the same time, though regional intrahepatic treatment is consistently associated with a relatively high rate of response, a significant proportion of patients still fail to respond. Therefore, further clinical trials should explore the effect of intra-arterial combinations of non-cross-resistant drugs, with possible synergistic effects. In addition, escalation of doses or a dose-dense approach may also contribute to decrease the rate of progression of the liver disease. Since hepatic metastases are the most common cause of death in patients with colorectal cancer, this approach may ultimately improve the prognosis of these patients.

### REFERENCES

Advanced Colorectal Cancer Meta-Analysis Project (1992) Modulation of fluorouracil by leucovorin in patients with advanced colorectal cancer: evidence in terms of response rate. *J Clin Oncol* **10**: 896–903

Cantrell JE Jr, Hart RD, Taylor RF and Harvey JH Jr (1987) Pilot trial of prolonged continuous infusion 5-fluorouracil and weekly cisplatin in advanced colorectal cancer. *Cancer Treat Rep* **71**: 615–618

Chang AE, Schneider PD, Sugarbaker PH, Simpson C, Culnane M and Steinberg SM (1987). A prospective randomized trial of regional versus systemic continuous 5-FUDR chemotherapy in the treatment of colorectal liver metastases. *Ann Surg* **206**: 685–693

Cox DR (1972) Regression models and life tables. *J Roy Stat Soc* **34**: 187–220

Douillard JY, Cunningham D, Roth AD, Navarro M, James RD, Karasek P, Jandik P, Iveson T, Carmichael J, Alakl M, Gruia G, Awad L and Rougier P (2000) Irinotecan combined with fluorouracil compared with fluorouracil alone as first-line treatment for metastatic colorectal cancer: a multicentre randomised trial. *Lancet* **355**: 1041–1047

Ensminger WD and Gyves JW (1983) Clinical pharmacology of hepatic arterial chemotherapy. *Semin Oncol* **10**: 176–183

Foster HJH and Berman MM (1977) Solid liver tumors. In: Ebert PA. (ed), *Major Problems in Clinical Surgery*, pp 209–234. Philadelphia: W.B. Saunders

Harmantas A, Rotstein LE and Langer B (1996) Regional versus systemic chemotherapy in the treatment of colorectal carcinoma metastatic to the liver. *Cancer* **78**: 1639–1645

Hohn DC, Stagg RJ, Friedman MA, Hannigan JF, Rayner A, Ignoffo RJ, Acord P and Lewis BJ (1989) A randomized trial of continuous intravenous versus hepatic intraarterial floxuridine in patients with colorectal cancer metastatic to

© 2001 Cancer Research Campaign the liver: the Northern California Oncology Group Trial. *J Clin Oncol* **7**: 1646–1654

Kaplan E and Meier P (1958) Nonparametric estimation for incomplete observations. *J Am Stat Assoc* **53**: 457–481

Kemeny N, Conti JA, Cohen A, Campana P, Huang Y, Shi WJ, Botet J, Pulliam S and Bertino JR (1994) Phase II study of hepatic arterial floxuridine, leucovorin, and dexamethasone for unresectable liver metastases from colorectal carcinoma. *J Clin Oncol* **12**: 2288–2295

Kemeny N, Daly J, Reichman B, Geller N, Botet J and Oderman P (1987) Intrahepatic or systemic infusion of FUDR in patients with liver metastases from colorectal carcinoma. A randomized trial. *Ann Intern Med* **107**: 459–465

Kemeney N and Ron I (1999) Hepatic arterial chemotherapy in metastatic colorectal patients. *Semin Oncol* **26**: 524–535

Kemeny N and Seiter K (1993) Treatment options for patients with metastatic colorectal cancer. In: Niederhuber JE (ed), *Current Therapy in Oncology*, pp 447–457. Mosby Year-Book: St Louis

Landis SH, Murray T, Bolden S and Wingo PA (1998) Cancer statistics, 1998. *CA Cancer J Clin* **48**: 6–29

Meta-Analysis Group in Cancer Reappraisal of hepatic arterial infusion in the treatment of nonresectable liver metastases from colorectal cancer (1996) *J Natl Cancer Inst* **88**: 252–258

Palmeri S, Gebbia V, Russo A, Gebbia N, Rostum Y and Rausa L (1992) Cisplatinum in combination with 5-fluorouracil and citrovorum factor in the treatment of advanced colorectal carcinoma. *Cancer Invest* **10**: 497–504

Patt YZ, Hoque A, Lozano R, Pazdur R, Chase J, Carrasco H, Chuang V, Delpassand ES, Ellis L, Curley S, Roh M and Jones DV (1997) Phase II trial of hepatic arterial infusion of fluorouracil and recombinant human interferon alpha-2b for liver metastases of colorectal cancer refractory to systemic fluorouracil and leucovorin. *J Clin Oncol* **15**: 1432–1438

Peto R, Pike MC, Armitage P, Breslow NE, Cox DR, Howard SV, Mantel N, McPherson K, Peto J and Smith PG (1977) Design and analysis of randomized clinical trials requiring prolonged observation of each patient. II. Analysis and examples. *Br J Cancer* **35**: 1–39

Pitot HC, Wender DB, O'Connell MJ, Schroeder G, Godlberg RM, Rubin J, Mailliard JA, Knost JA, Ghosh C, Kirschling RJ, Levitt R and Windschitz HE (1997) Phase II trial of irinotecan in patients with metastatic colorectal carcinoma. *J Clin Oncol* **15**: 2910–2919

Rougier P, Bugat R, Douillard JY, Culine S, Suc E, Brunet P, Becouarn Y, Ychou M, Marty M, Extra JM, Bonneterre J, Adenis A, Seitz JF, Ganem G, Namer M, Conroy T, Negrier S, Merrouche Y, Burki F, Mousseau M, Herait P and Mahjoubi M (1997) Phase II study of irinotecan in the treatment of advanced colorectal cancer in chemotherapy-naive patients and patients pretreated with fluorouracil-based chemotherapy. *J Clin Oncol* **15**: 250–260

Schabel FM, Trader MW, Laster WR, Corbett TH and Griswold DP Jr (1979) Cis-platinum: combination chemotherapy and cross-resistant studies with tumors of mice. *Cancer Treat Rep* **63**: 1459–1473

Scheithauer W, Rosen H, Schiessel R, Schuller J, Karall M, Ernst F, Sebesta C, Kornek J, Hentschel E and Marczel A (1991) Treatment of patients with advanced colorectal cancer with cisplatin, 5-fluorouracil and leucovorin. *Cancer* **67**: 1294–1298

*© 2001 Cancer Research Campaign*
